# A TNM Staging System for Nasal NK/T-Cell Lymphoma

**DOI:** 10.1371/journal.pone.0130984

**Published:** 2015-06-22

**Authors:** Zheng Yan, Hui-qiang Huang, Xiao-xiao Wang, Yan Gao, Yu-jing Zhang, Bing Bai, Wei Zhao, Wen-qi Jiang, Zhi-ming Li, Zhong-jun Xia, Su-xia Lin, Chuan-miao Xie

**Affiliations:** 1 Department of Medical Oncology, Sun Yat-sen University Cancer Center, Guangzhou, Guangdong, China; 2 Stage Key Laboratory of Oncology in South China, Guangzhou, Guangdong, China; 3 Collaborative Innovation Center for Cancer Medicine, Guangzhou, Guangdong, China; 4 Department of Radiation Oncology, Sun Yat-sen University Cancer Center, Guangzhou, Guangdong, China; 5 Department of Hematology, Sun Yat-sen University Cancer Center, Guangzhou, Guangdong, China; 6 Department of Pathology, Sun Yat-sen University Cancer Center, Guangzhou, Guangdong, China; 7 Medical Imaging Department, Sun Yat-sen University Cancer Center, Guangzhou, Guangdong, China; School of Medicine, Fu Jen Catholic University, TAIWAN

## Abstract

Ann Arbor stage has limited utility in the prognostication and treatment decision making in patients with NK/T-cell lymphoma (NKTCL), as NKTCL is almost exclusively extranodal and the majority is localized at presentation for which radiotherapy is the most important treatment and local invasiveness is the most important prognostic factor. In this study, we attempted to establish a TNM (Tumor-Node-Metastasis) staging system for nasal NKTCL (N-NKTCL). The staging rules of other head and neck cancers were used as reference along with the data of our 271 eligible patients. The primary tumor was classified into T1 to T4, and cervical lymph node metastasis was classified into N0 to N2 according to the extent of involvement. Any lesions outside the head and neck were classified as M1. N-NKTCL thereby was classified into four stages: stage I comprised T1-2N0M0; stage II comprised T1-2N1M0 and T3N0M0; stage III comprised T3N1M0, T1-3N2M0, and T4N0-2M0; and stage IV comprised TanyNanyM1. This staging system showed excellent performance in prognosticating survival. In the current series, the 5-year survival rates of patients with stages I, II, III, and IV N-NKTCL were 92%, 64%, 23%, and 0, respectively. Moreover, the predictive value of several currently used factors was abrogated in the presence of the TNM stage. The TNM staging system is highly effective in stratifying tumor burden and survival risk, which may have significant implications in the treatment decision making for patients with N-NKTCL.

## Introduction

Tumor staging plays a critical role in treatment decision making and outcome assessment for patients with various cancers, especially in settings where local-regional treatment is definitive. Meanwhile, in virtually all kinds of cancers, stage is the strongest prognostic factor. Thus, each comprehensive diagnosis for cancer contains a stage as a quantitative diagnosis indicating tumor burden and prognosis.

NK/T-cell lymphoma (NKTCL) has been generally classified into nasal NKTCL (N-NKTCL) involving the upper aerodigestive tract (UAT) and extranasal NKTCL (E-NKTCL) including primary tumors outside the UAT [1]. N-NKTCL accounts for approximately 80% in newly diagnosed cases, in which approximately 80% of the cases are localized [2, 3]. The disease was not well understood in the past due to its rarity in the developed Western countries. In recent years, it has attracted an increasing amount of attention and consensus on treatment was gradually established. However, there is no standard staging system thus far. The Ann Arbor (AA) staging system that was originally designed for Hodgkin Lymphoma is conventionally used for NKTCL. However, this staging method has limited utility in the prognostication and treatment decision making in patients with NKTCL, as the NKTCL is almost exclusively extranodal and the majority is localized at diagnosis for which radiotherapy is the most important treatment and local invasiveness is the most important prognostic factor [4, 5], but the AA system does not take into account the tumor size and invasion to contiguous structures. The AA stage I NKTCL actually consists of a group of highly heterogeneous disease with various aggressiveness and prognosis that should be treated differently. To compensate for the insufficiency of AA stage in prognostication, an increasing number of prognostic factors have been identified, suck as the number of involved extranodal sites, lactate dehydrogenase (LDH) level, B symptoms, performance status (PS), local tumor invasiveness (LTI), regional lymph node metastasis (RLNM), and pretreatment plasma EBV-DNA number [5–8]. These factors, representing tumor burden in some way, are important, however excessive and confusing. Thus, an appropriate staging system that can effectively stratify tumor burden and survival risk is urgently needed, thereby helping guide treatment decisions.

Some investigators classified NKTCL into limited disease (AA stage I/II N-NKTCL without LTI) and extensive disease (stage I/II with LTI or stage III/IV disease of N-NKTCL, and E-NKTCL) [9]. However, this classification has limitations: (1) limited disease includes tumor with RLNM which has been documented as a poor prognosis predictor [6]; (2) extensive disease comprises a group of highly heterogeneous diseases, some of which are localized that can be cured by radiotherapy or chemo-radiotherapy and some others have distant metastasis that could only be treated with chemotherapy; and (3) some E-NKTCLs are localized with favorable prognosis. Besides, a T-staging system originally designed for sinonasal B-cell lymphoma was recommended for N-NKTCL by some investigators [10, 11], but this system only has the T classification for nasal cavity lymphoma.

According to reports from other researchers and to our own experience, we concluded that N-NKTCL shares several similar clinical features with solid tumors: (1) it regularly derives from the mucosa of the UAT, rather than lymph nodes or lymphatic organs; (2) the majority of newly diagnosed cases are localized for which radiotherapy, rather than chemotherapy, is the most important treatment; (3) LTI and RLNM are the strongest prognostic factors for early stage disease; and (4) when the disease disseminates beyond the primary site and regional node, it may be treatable, but it is probably not curable. These features prompted us to adopt the solid tumor staging rules for N-NKTCL. In the present study, we tried to establish a TNM staging system for N-NKTCL based on our data and the staging rules of other head and neck cancers. The TNM staging system showed excellent performance in prognosticating survival and may help guide treatment decisions.

## Patients and Methods

### Ethics statement

This study was approved by the Institutional Review Board of Sun Yat-Sen University Cancer Center, and written informed consent was obtained from all patients at the time of first admission. Additionally, this study was conducted in accordance with the Helsinki Declaration.

### Patients

The data of patients with NKTCL diagnosed and treated at Sun Yat-sen University Cancer Center in China from January 2001 to June 2013 were retrospectively collected. To maintain the accuracy and reliability of the data, stringent exclusion criteria were employed. Patients who had suspicious pathological diagnosis, incomplete imaging data to detail lesions at baseline, and who died of other causes were excluded. Since radiotherapy plays a critical role in treating early stage N-NKTCL and chemotherapy may benefit patients at advanced stage, patients with local/local-regional disease who did not receive radiotherapy if medically possible and patients with advanced disease who did not receive any kind of chemotherapy were excluded. Given the possibility that some Chinese hospitals have inadequate experience in treating this disease, patients who were previously treated in other hospitals were excluded. Patients with primary E-NKTCL were not included in this study.

### Methods

The clinical characteristics, treatment, and follow-up information of eligible patients were extracted from the medical records and the follow-up system. The anatomic sites and structures involved by the tumor in each patient were recorded by reviewing the patients’ imaging reports, including CT, MRI, and positron emission tomography computed tomography which were attained before treatment. The image was reviewed once again by two radiologists if the description in the report was ambiguous. Physical examination information was also used in data collection.

To establish the T classification, several factors were taken into consideration: the staging rules of other head and neck cancers [the T-staging system proposed for sinonasal B-cell lymphoma (Table 1) [10], the 6th edition of UICC/AJCC TNM staging system for nasopharyngeal carcinoma (NPC) (Table 2) [12], and the UICC/AJCC TNM system for nasal cavity and paranasal sinus cancers [13]; the anatomical distance from the involved tissues and structures to the primary site; and the survival time of patients with the same extent of disease. For N classification, we referred to the staging rules of other head and neck cancers and compared the survival time of patients with different patterns of RLNM. The prognosis of patients with N-NKTCL and extranasal dissemination is extremely poor. Thus, any kind of lesions outside the head and neck was regarded as distant metastasis, namely M1. Otherwise, it was classified as M0.

**Table 1:** The T-staging system proposed for sinonasal B-cell lymphoma.

Stage	Definition
T1	Confinement to the nasal cavity.
T2	Extension to the maxillary sinus, anterior ethmoid sinus, or hard palate.
T3	Extension to posterior ethmoid sinus, sphenoidal sinus, orbit, superior alveolar bone, cheeks, or superior buccinators space.
T4	Involvement of the inferior alveolar bone, inferior buccinators space, infratemporal fossa, nasopharynx, or cranial fossa.

**Table 2:** The T classification of the 6th edition of UICC/AJCC TNM staging system for nasopharyngeal carcinoma.

Classification	Definition
T1	Nasopharynx
T2	Parapharyngeal extension, oropharynx and/or nasal cavity
T3	Skull base, bony structures and/or paranasal sinuses
T4	Intracranial extension and/or cranial nerves, infratemporal fossa, hypopharynx, orbit or masticatory space

After the establishment of the T, N, and M classifications, various combinations of these classifications were tried to determine the best stage grouping method by observing the distribution of the survival curves stratified by the newly designed staging system.

To test the applicability of the new staging system, the prognostic value of this system and that of the currently used AA system were compared, and the prognostic value was also tested on progression-free survival (PFS). Meanwhile, the commonly used prognostic factors for patients with NKTCL were tested by univariate and multivariate analyses in the presence of the TNM stage.

### Statistical analysis

Statistical analyses were performed using SPSS version 18.0. Overall survival (OS) was measured from the date of diagnosis to the date of death due to tumor or the last follow-up, and PFS was calculated from the date of diagnosis to the date of progression or relapse or the last follow-up. OS and PFS curves were derived using the Kaplan-Meier method. OS and PFS rates were calculated using the life table method. The differences between survival curves were calculated using the log-rank test. Multivariate analysis was performed using the Cox model. *P* ≤ 0.05 was considered statistically significant, and all the *P* values correspond to two-sided significance tests.

## Results

### Eligible patients and the characteristics

We screened 405 patients with NKTCL from our database, 134 of which were excluded from the analysis for the following reasons: 52 patients with local/local-regional disease did not receive radiotherapy; 42 patients had primary E-NKTCL; 17 patients (13 were AA stage I/II and 4 were stage III/IV) did not receive anticancer treatment; 10 patients were previously treated in other hospitals; 7 patients had incomplete imaging data; 2 patients died of other diseases; and the pathological diagnosis for 4 patients was ambiguous. The remaining 271 eligible patients were included in the analysis.

The data of phenotypic markers of the tumor were unavailable in patients diagnosed in early days. In the data available, the ratio of positive expression was as follows: 91.7% (231/252) for CD3ε, 91.3% (232/254) for CD56, 97.6% (165/169) for granzyme B, 82.0% (109/133) for perforin, 98.0% (198/202) for TIA-1, and 98.9% (187/189) for EBER. The clinical characteristics of the 271 patients are available in Supporting Information (S1 File). According to the AA staging system, 190 (70.1%), 46 (17.0%), 9 (3.3%), and 26 (9.6%) patients were classified into stages I, II, III, and IV, respectively. The anatomic sites and structures involved by the lymphoma are listed in Table 3.

**Table 3:** Tumor-invaded anatomic sites and structures.

Anatomic site	No. (%)
Primary sites of tumor*	
Nasal cavity	219 (80.8)
Nasopharynx	32 (11.8)
Oropharynx	11 (4.1)
Oral cavity	6 (2.2)
Hypopharynx	3 (1.1)
Locally invaded structures	
Maxillary sinus	59 (21.8)
anterior ethmoid sinus	53 (19.6)
Skull base	25 (9.3)
Posterior ethmoid sinus	39 (14.4)
Nosewing	35 (12.9)
Palate	29 (10.7)
parapharyngeal space	19 (7.0)
Inferior and medial wall of the socket	19 (7.0)
Cheeks	16 (5.9)
Sphenoid sinus	15 (5.5)
Alveolar bone	11 (4.1)
Socket other than the inferior and medial walls	6 (2.2)
Frontal sinus	8 (3.0)
Pterygoid muscles	9 (3.3)
Masticator space excluding pterygoid muscles	6 (2.2)
Intracranial extension	5 (1.8)
Regional lymph node	56 (20.7)
Distant metastases	
Non-regional lymph node	15 (5.5)
Skin	12 (4.4)
Lung	12 (4.4)
Liver	7 (2.6)
Spleen	6 (2.2)
Reproductive organs	5 (1.8)
Kidney	4 (1.5)
Adrenal gland	4 (1.5)
Pancreas	4 (1.5)
Digestive tract	3 (1.1)
Bone	3 (1.1)
Bone marrow	1 (0.4)

*When the tumor invaded more than one site of the upper aerodigestive tract, the predominant site was considered to be the primary origin.

### Treatment, prognosis, and survival

Except for the 15 patients with very early stage disease that received radiation monotherapy, 256 patients received first-line chemotherapy. The median number of chemotherapy cycles was 3 (range, 1–10 cycles). The chemotherapy regimens included EPOCH (etoposide, doxorubicin/epirubicin, vincristine, cyclophosphamide, and prednisolone; 76 patients), L/P-Gemox (L-asparaginase/Pegaspargase, gemcitabine, and oxaliplatin; 61 patients), CHOP (or CHOP-like) (cyclophosphamide, doxorubicin/epirubicin, vincristine, and prednisolone, 89 patients), L/P-CHOP (L-asparaginase/Pegaspargase, CHOP; 7 patients), Gemox (gemcitabine, oxaliplatin; 5 patients), PGM (pegaspargase, gemcitabine, and methotrexate; 7 patients), VDL (vincristine, dexamethasone, and L-asparaginase; 4 patients), and other regimens in 7 patients. Of the 230 patients with local/local-regional disease, 15 patients with very early stage tumor received radiation monotherapy, while 213 patients received combined chemo-radiotherapy. The remaining two patients with extensive local infiltration that cannot be treated by radiation received chemotherapy alone. In the 41 patients with distant metastasis, 9 patients received radiotherapy in addition to chemotherapy. The radiotherapy was interrupted in 3 patients due to disease progression during treatment. In the 235 patients who completed the planned radiotherapy, the median dose of radiation was 54.6 Gy (range, 36–64 Gy) for the primary tumor. Ninety-three patients had neck radiation with a median dose of 46 Gy (range, 26–60 Gy).

By June 2014, 84 patients (31.0%) have died of cancer; 159 patients (59.8%) were alive; and 25 patients (9.2%) were lost to follow-up. The median follow-up time was 37.7 months (range, 1.2–159.7 months). The 3- and 5-year OS rates were 70% and 64%, respectively.

### The proposed T classification

N-NKTCL may derive from the entire mucosa of the UAT, from nasopharynx to hypopharynx. Therefore, the T classification was considered separately according to the primary site.

For NKTCL deriving from the nasal cavity, we adopted the main ideas of the T-staging system proposed for sinonasal B-cell lymphoma (Table 1) [10] with a few modifications. Some NKTCLs tend to creep along the surface of mucosa, the prognosis of which is favorable if no surrounding structures are involved, so we classified the involvement of nasopharynx into T2 and the involvement of parapharyngeal space and pterygoid muscles into T3. The prognosis is different between the patients with the involvement of the orbital wall and the involvement of the intraorbital organs. Besides, in the UICC/AJCC TNM system for nasal cavity and paranasal sinus cancers, the involvement of the side or bottom wall of the eye socket was categorized into T3, while the invasion of the front part of the eye socket was classified into T4 [13]. Thus, we classified the involvement of the medial or inferior wall of the eye socket into T3 and the involvement of the other parts of the eye socket into T4. Tumor-related palate perforation generally results in serious dysfunction and poor prognosis, so it was classified into T4.

For NKTCL deriving from the nasopharynx, we adopted the T classification of the 6th edition of UICC/AJCC staging system for NPC [12], as they share the similar growth patterns and treatment strategies.

NKTCLs originating from the oral cavity, oropharynx, and hypopharynx are extremely rare, and generally can be diagnosed at very early stage. The T classifications were proposed based on the growth pattern of the tumor, the anatomy of the tumor site, and the available data in our study.

**Fig 1:**
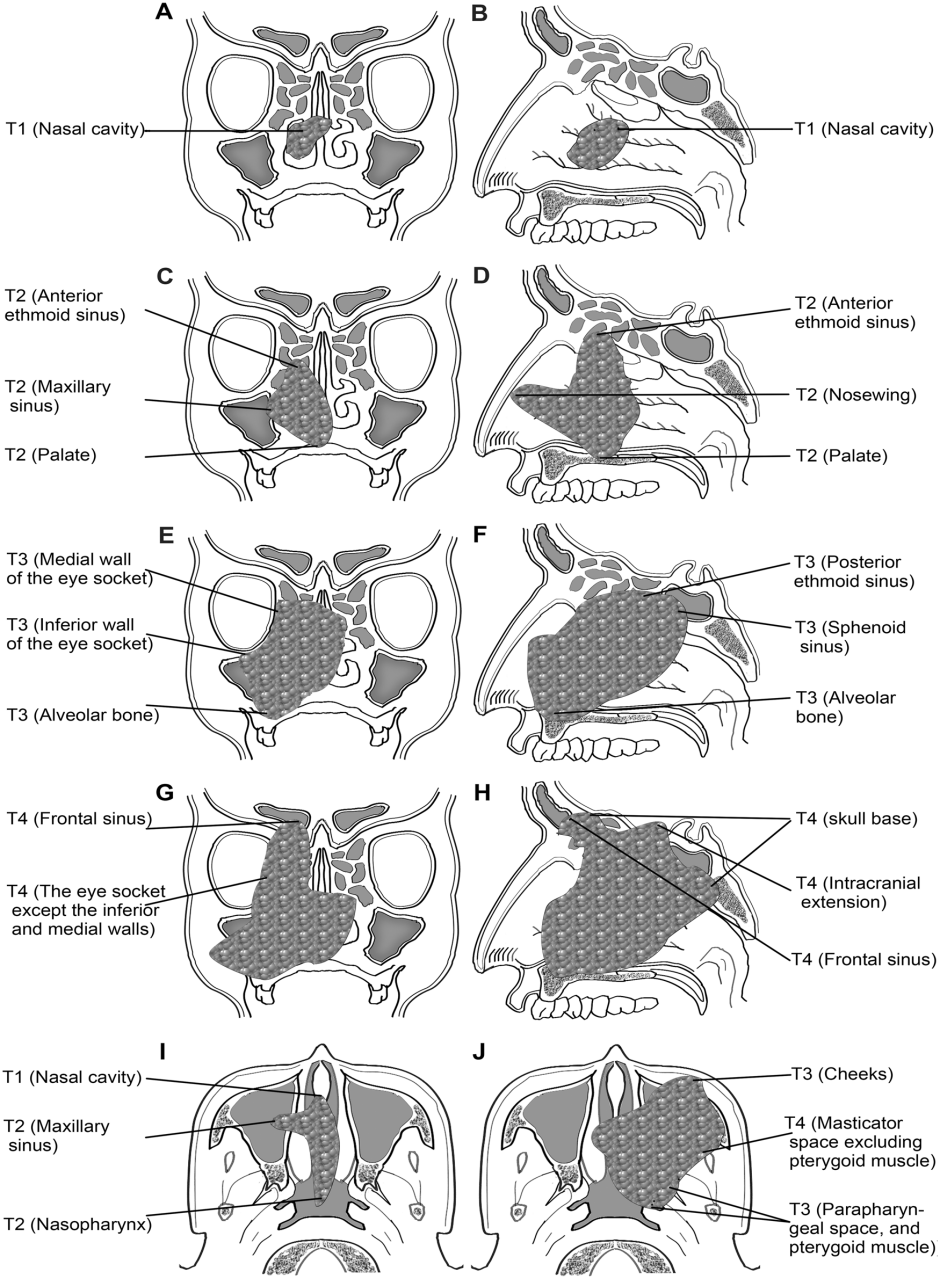
Veiws of definition of T classifications for tumor originating from the nasal cavity. A, C, E, and G are coronal planes; B, D, F, and H are sagittal planes; I and J are tansverse planes.

**Fig 2:**
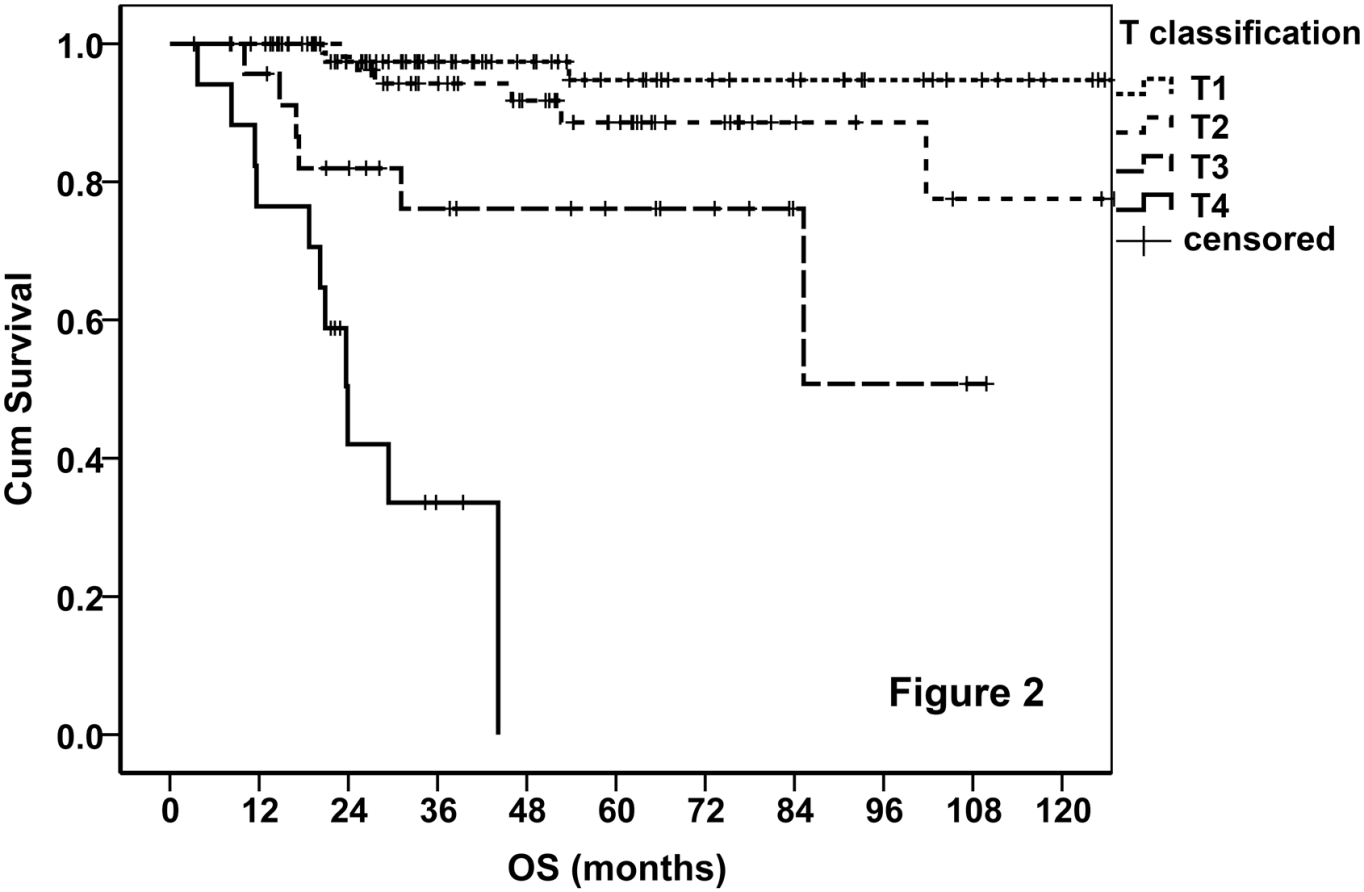
Survival curves stratified by the proposed T classification.

The finally proposed T classifications are listed in Table 4. Because the T classification for tumor from the nasal cavity is somewhat complicated, it is also demonstrated by atlas (Fig 1). The OS curves stratified by the T classifications are demonstrated in Fig 2, in which only the data of localized disease were analyzed to avoid the interference of regional and distant metastasis. It indicated that T3 was a moderate adverse predictor for survival, while T4 was a strong one.

**Table 4:** The proposed T classifications for the primary tumor.

Primary tumor site	T classification	Definition
Nasal cavity	T1	Nasal cavity
	T2	Maxillary sinus, anterior ethmoid sinus, nosewing, palate, nasopharynx
	T3	Posterior ethmoid sinus, cheeks, alveolar bone, inferior or medial wall of the eye socket, sphenoid sinus, parapharyngeal space, pterygoid muscles
	T4	Frontal sinus, the eye socket except the inferior and medial walls, masticator space excluding pterygoid muscles, skull base, intracranial extension, cranial nerve, perforated palate
Nasopharynx	T1	Nasopharynx
	T2	Parapharyngeal space
	T3	Skull base, pterygoid muscles, paranasal sinuses
	T4	Cranial nerves, intracranial extension, masticatory space excluding pterygoid muscles, orbit
Oral cavity	T1	Oral cavity
	T2	Palate, alveolar bone, oropharynx
	T3	Maxillary sinus, skin, hypopharynx
	T4	More extensive invasion, perforation
Oropharynx Hypopharynx	T1	Oropharynx or hypopharynx
	T2	Oropharynx and hypopharynx, palate
	T3	Bone, cartilage, and skin around oropharynx, tumor-related dysphagia
	T4	More extensive invasion, tumor-related dyspnea, perforation

### The proposed N classification

**Fig 3:**
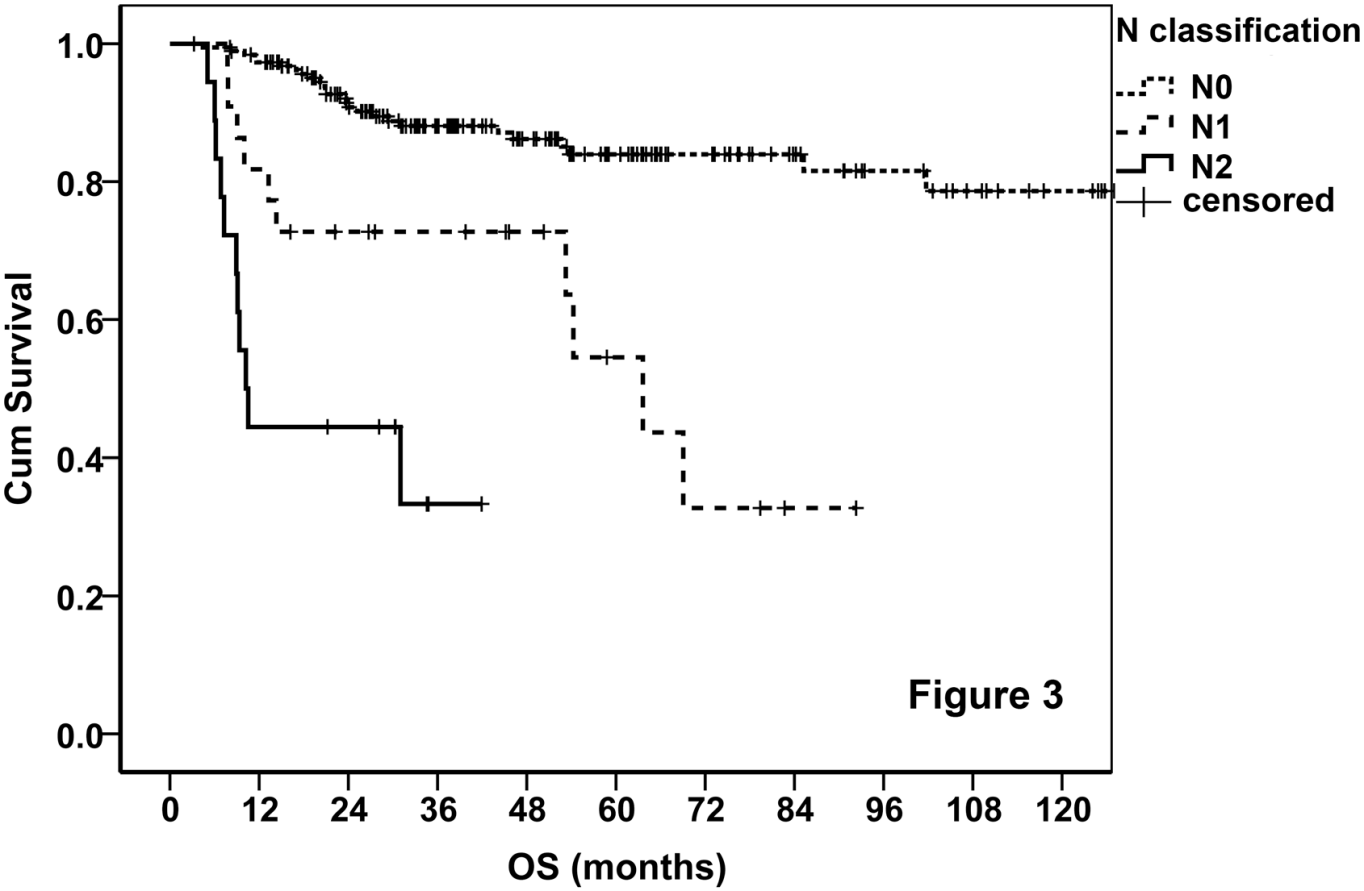
Survival curves stratified by the proposed N classification.

For most kinds of head and neck cancers, N classification considers the side (unilateral or bilateral) and the number (one or more) of RLNM, and the size of the largest lymph node (3 or 6 cm). For NKTCL, the clinical relevance of these factors is unknown, so the prognostic value of RLNM was explored by survival analysis at first. To avoid the interference of distant metastasis, 41 patients with M1 disease were excluded. In the 230 patients with local/local-regional disease, 22 and 18 patients had unilateral and bilateral RLNM, respectively. The lymph node with a diameter > 3 cm was observed only in two patients, making it impossible to analyze the size of lymph node. The number of lymph nodes was not analyzed either. We classified the 230 patients into 3 groups: patients with no, unilateral, and bilateral RLNM. As shown in Fig 3, the prognosis of the patients in the 3 groups was significantly different (*P* < 0.001). Unilateral RLNM was a moderate adverse prognosticator, while bilateral RLNM was a strong one. Thus, no, unilateral, and bilateral RLNM were classified as N0, N1, and N2, respectively.

### The proposed stage group

The NKTCL of the 41 patients with distant metastasis (M1) was grouped into stage IV regardless of the T and N classifications. Of the 230 cases with local/local-regional disease, the prognosis was excellent in patients with T1/T2 and N0 disease (Fig 2), so T1-2N0M0 were grouped into stage I (stage IA, T1N0M0; and stage IB, T2N0M0). Given the moderate adverse prognosis of patients with T3 and N1 disease, T3N0M0 and T1-2N1M0 were grouped into stage II. However, when a tumor had both T3 and N1, it was grouped into Stage III. Both T4 and N2 were strong adverse prognosticators (Figs 2 and 3), hence, T4NanyM0 and TanyN2M0 were grouped into stage III. These stage groups are concluded in Table 5. Base on the proposed stage groups, the stage distribution of the disease in the 230 patients with local/local-regional involvement is listed in Table 6.

**Table 5:** The proposed stage groups.

Stage group		T classification	N classification	M classification
I	IA	T1	N0	M0
	IB	T2	N0	M0
II		T3	N0	M0
		T1-2	N1	M0
III		T3	N1	M0
		T1-3	N2	M0
		T4	N0-2	M0
IV		T any	N any	M1

**Table 6:** The stage distribution of the disease in the 230 patients with local/local-regional involvement.

Stage	N0	N1	N2	Total
T1	88	4	4	96
T2	61	7	4	72
T3	24	8	8	40
T4	17	3	2	22
Total	190	22	18	230

**Fig 4:**
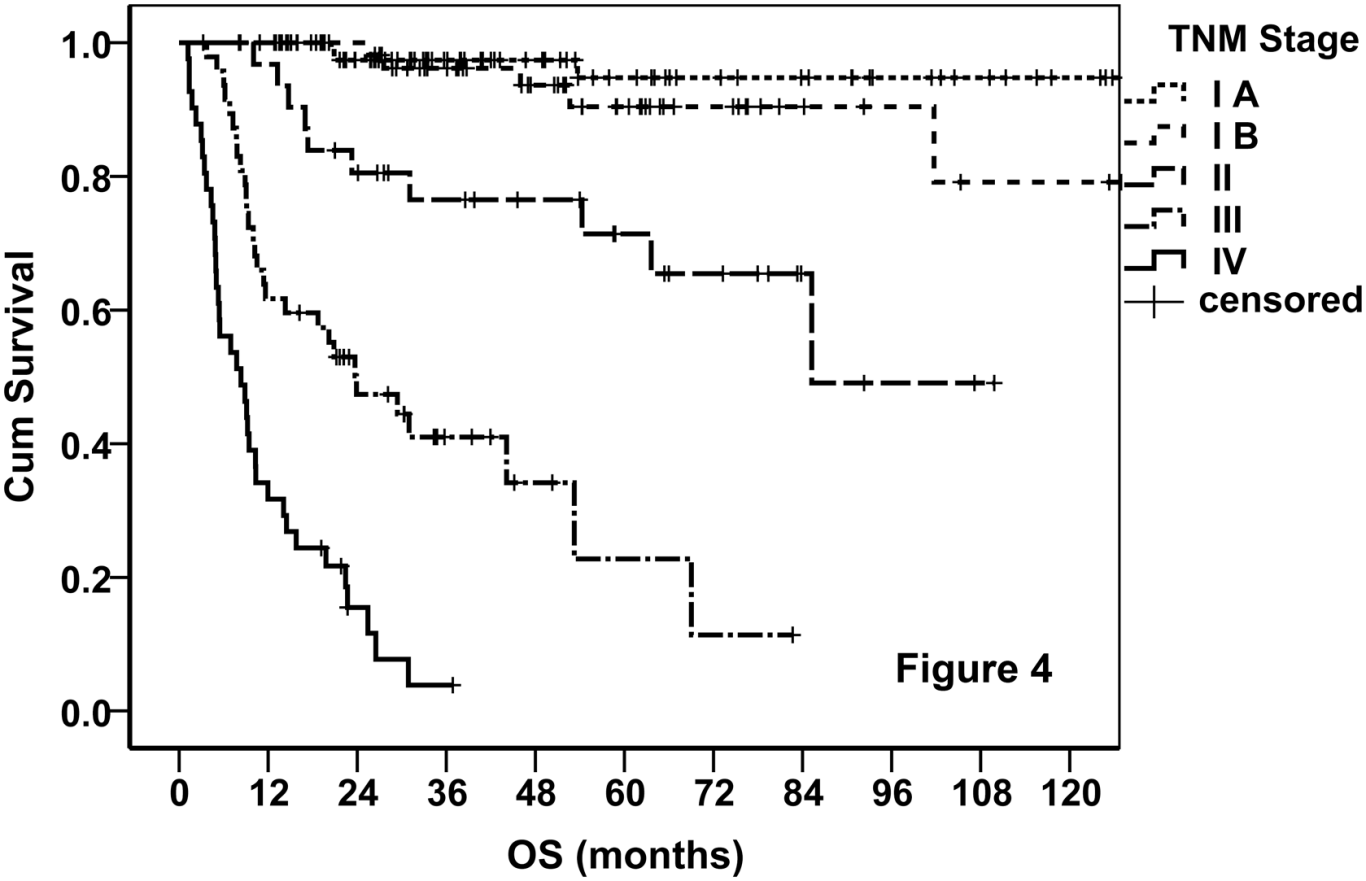
OS curves of the entire cohort stratified by the proposed TNM staging system.

**Fig 5:**
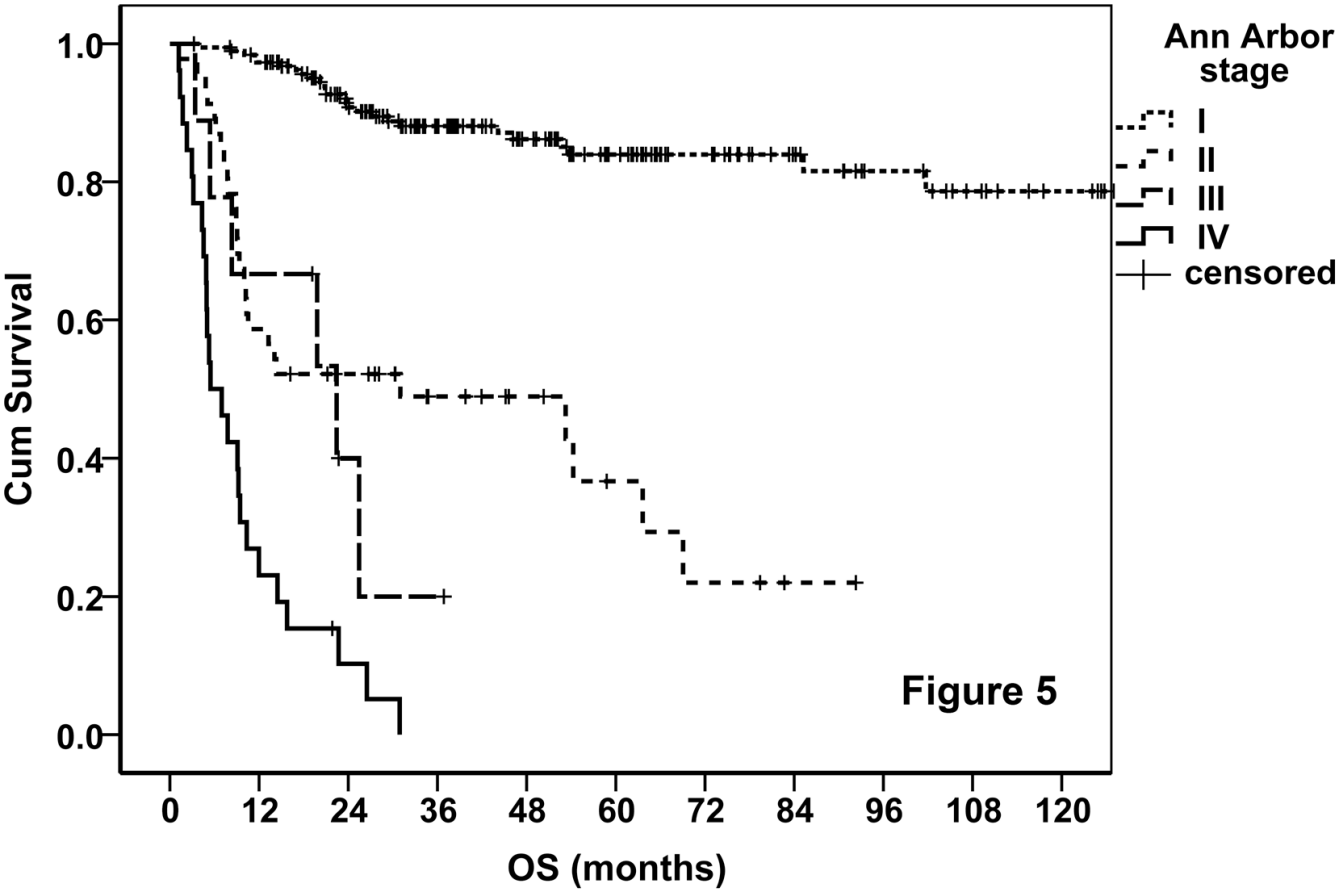
OS curves of the entire cohort stratified by AA system.

**Fig 6:**
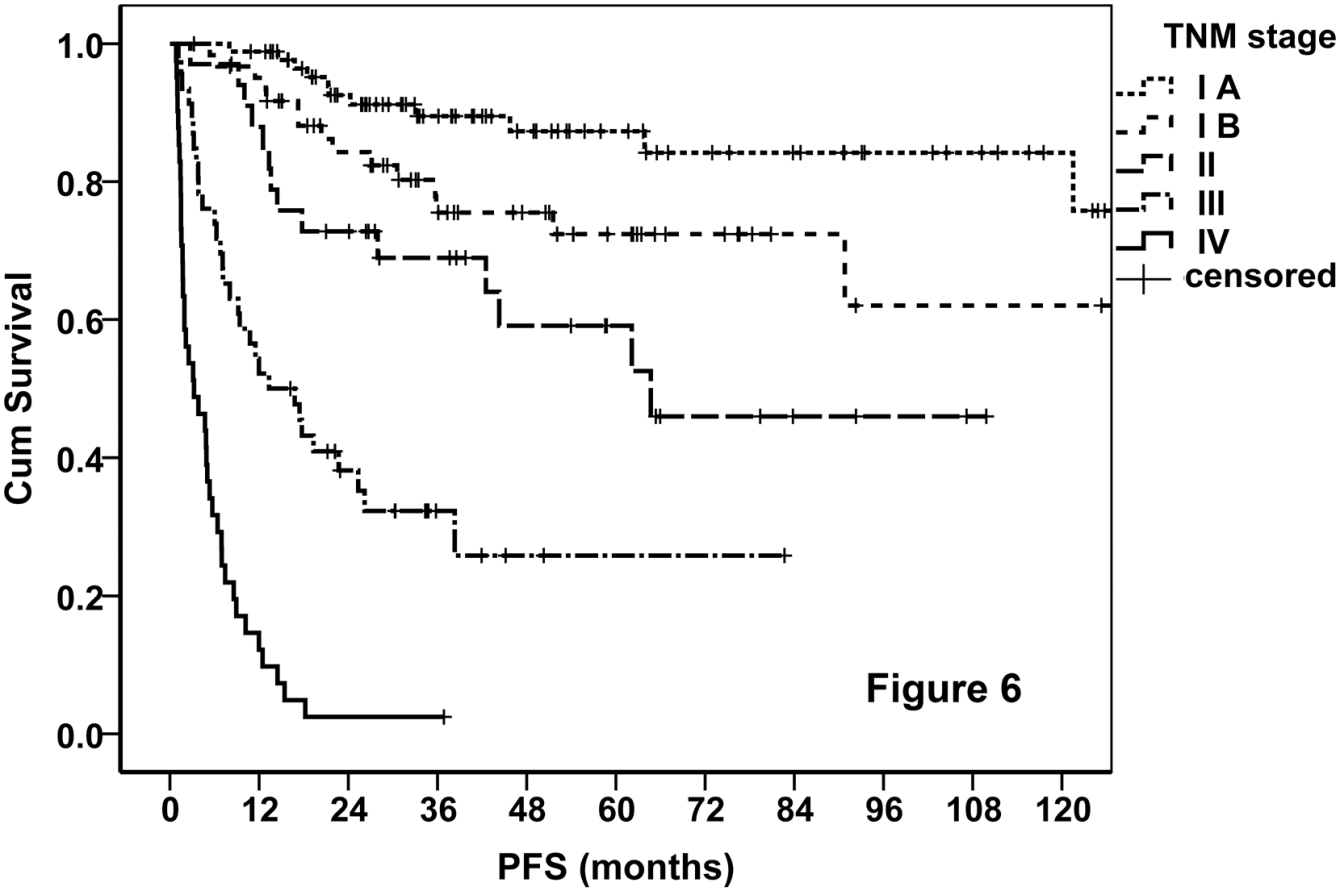
PFS curves of the entire cohort stratified by the TNM staging system.

Finally, 88 (32.5%), 61 (22.5%), 35 (12.9%), 46 (17%), and 41 (15.1%) patients were classified into stages IA, IB, II, III, and IV, respectively. The OS curves of the entire cohort stratified by the proposed TNM stage were demonstrated. As shown in Fig 4, the new staging system performed excellently in prognostication, and it was much better than AA system (Fig 5). We tried various combinations of the T and N classifications, but none of these showed better results. The TNM staging system also worked very well when it was applied on PFS (Fig 6). According to the new staging system, the 5-year OS rates were 92%, 64%, 23%, and 0 for patients with stages I, II, III, and IV disease, respectively, and the corresponding 5-year PFS rates were 79%, 45%, 23%, and 0, respectively. The prognosis of stage IB disease was slightly poorer when compared with stage IA disease (Table 7). According to the AA stage, the 5-year OS rates were 84%, 24%, 0, and 0, and the 5-year PFS rates were 70%, 22%, 0, and 0, in patients with stages I, II, III, and IV disease, respectively.

**Table 7:** The prognosis of patients with different stage disease classified by the TNM staging system.

TNM Stage	No. (%)	Median OS, mo	5-year OS, %	5-year PFS, %
I	149 (55.0)	NR	92	79
IA	88 (32.5)	NR	95	84
IB	62 (22.5)	NR	89	73
II	35 (12.9)	85.2	64	45
III	46 (17.0)	23.9	23	23
IV	41 (15.1)	8.3	0	0
Total	271 (100)	NR	64	54

Abbreviations: OS, overall survival; PFS, progression-free survival; NR, not reached.

### Prognostic factors for survival

Univariate analysis demonstrated that all of the commonly used factors, including B symptoms, LDH level, AA stage, RLNM, PS, LTI, pretreatment EBV-DNA level, and age, had statistically significant prognostic value. Besides, non-tumor-infiltrated splenomegaly that is very common in patients with NKTCL was also an adverse predictor. In multivariate analysis, however, only TNM stage and age were independent predictors (Table 8).

**Table 8:** Prognostic factors for survival in univariate and multivariate analyses.

Parameters	Univariate analysis	Multivariate analysis		
	*P*	RR	95% CI	*P*
B symptoms (yes)	0.001	0.858	0.505–1.457	0.571
LDH level (> normal limit)	<0.001	0.918	0.519–1.623	0.768
AA stage (III/IV)	<0.001	0.485	0.191–1.233	0.128
Regional lymph node (yes)	<0.001	1.505	0.909–2.494	0.112
ECOG PS (> 1)	<0.001	1.590	0.765–3.306	0.214
Local invasiveness (yes)	<0.001	1.690	0.899–3.176	0.103
EBV-DNA (> 500 copies/mL)*	0.003	1.727	0.560–5.323	0.342
Age (> 60 years)	0.003	2.188	1.178–4.063	0.013
Splenomegaly (yes)^†^	0.005	1.331	0.845–2.096	0.217
TNM stage				
II	<0.001	3.335	1.244–8.944	0.017
III	<0.001	15.804	6.112–40.869	<0.001
IV	<0.001	99.775	29.307–339.685	<0.001

*Data were available in 128 patients. When this variable was analyzed, the cases without data were excluded.

^†^Splenomegaly indicates splenic enlargement without abnormal density lesions, which was not considered as tumor infiltration.

Abbreviations: LDH, lactate dehydrogenase; AA, Ann Arbor; ECOG PS, Eastern Cooperative Oncology Group performance status.

## Discussion

Unlike other kinds of lymphomas, NKTCL is almost exclusively extranodal, in which the vast majority originates from the mucosa of the UAT and tends to infiltrate the surrounding tissues and structures. The disease is localized in the majority of patients at presentation. At advanced stage, the tumor may disseminate to any organ of the body. These growth patterns are similar to solid tumors.

Radiotherapy was widely recognized as the most important treatment for early stage N-NKTCL [14–20]. The expression of P-glycoprotein on NKTCL cells was blamed for chemotherapy resistance to anthracycline-based regimens, but this is not the only cause. The majority of the patients who achieved complete remission (CR) with chemotherapy alone eventually experienced relapse. The 5-year OS rate of AA stage I/II NKTCL patients treated with chemotherapy alone was only 13% to 35% [19–21]. In recent years, asparaginase (ASP) (or pegaspargase)-containing regimens showed prominent activity against NKTCL in a number of reports when historically compared with non-ASP-containing regimens and they are recommended by guidelines [18, 22]. However, whether ASP-containing chemotherapies could improve OS is in question due to the lack of contemporaneous comparison. In this cohort, the OS of patients treated with ASP-containing chemotherapies was only marginally superior to that of patients treated with non-ASP-containing chemotherapies (*P* = 0.057) (S2 File). New regimens in developing may improve outcomes to some extent, however, the role of radiotherapy cannot be replaced. Moreover, LTI and RLNM are documented to be the most important prognostic factors for early stage NKTCL [5, 6]. These features warrant the adoption of T and N staging for N-NKTCL. Previous study has demonstrated that T-stage based on the extent of stage I lymphoma of nasal cavity and paranasal sinuses was the strongest predictor of outcome [23].

The prognosis of patients with advanced stage NKTCL is dismal, with a median survival time of 6 to 12 months in previous reports [24], which may be partly attributed to the suboptimal treatment. New treatment approaches, such as ASP-containing chemotherapy and hematopoietic stem-cell transplantation (HSCT) seemed promising for such patients [25]. High-dose chemotherapy and HSCT resulted in a relatively high proportion of long-term survivors in several retrospective studies with small sample size [26–29]. However, the encouraging results may be largely attributed to selection bias and were complicated by early stage cases. In several prospective trials, however, the outcomes were unsatisfactory. In the NK-cell tumor study group study [30], 38 newly diagnosed stage IV, relapsed, or refractory NKTCL patients were treated with SMILE (L-asparaginase, methotrexate, ifosphamide, etoposide, and dexamethasone) chemotherapy which is regarded as one of the most effective regimens and with HSCT in selected patients, resulting in a CR rate of 45%. With such an aggressive treatment, the 1-year OS rate was 55% in the entire group and it was 45% in the newly diagnosed stage IV patients. Although the median survival time of the newly diagnosed stage IV patients was not reported, it was estimated to be < 1 year according to the 1-year OS rate. The 3-year OS rate of the entire group was reported to be 50% in 2013 [31], which seemed encouraging. However, in this study, 11 patients had AA stage I/II disease and 19 patients underwent HSCT. In the phase II study from France [32], the median survival time was 12.2 months in 19 relapsed/refractory NKTCL patients (12 patients at AA stage I/II) treated with the AspaMetDex regimen (L-asparaginase, methotrexate, and dexamethasone) and with HSCT in two patients. In a more recent study focusing only on stage IV NKTCL, The median survival time was only 10.6 months in 27 newly diagnosed patients who were treated with the SMILE regimen and autologous HSCT [33]. According to our experience, ASP-based chemotherapies can lead to rapid tumor regression in the majority of advanced NKTCL, however, relapse and progression are almost inevitable. In the present study, the median survival time of stage IV N-NKTCL was 8 months, with 1- and 3-year OS rates of 15% and 4%, respectively. The survival time of the unique long-term survivor lasted for 36.9 months by the time of the last follow-up, while this patient had two lesions in the nasal cavity and spleen, and both lesions were treated with radiotherapy. Taken together, it seems that disseminated N-NKTCL cannot be cured by conventional chemotherapy. This feature is also similar to solid tumors. The efficacy of HSCT needs to be evaluated in randomized trials with larger sample size. Given these evidences, we believe it is reasonable to adopt the TNM staging for N-NKTCL. Thus, we used the staging rules of other head and neck cancers as reference, classifying the primary tumor into T1 to T4 and the RLNM into N0 to N2, according to the extent of tumor involvement, while classifying tumor with any lesion outside the UAT into M1. As shown in Figs 4 and 6, the TNM staging system exhibited excellent performance in prognostication. The commonly used prognostic factors, including LDH level, B symptoms, PS, LTI, RLNM, and EBV-DNA level [5–8], were also predictive in the present study. Non-tumor-infiltrated splenomegaly that is very common in patients with NKTCL but was never reported before was also an adverse predictor revealed by univariate analysis. Interestingly, the predictive value of these factors was abrogated in multivariate analysis in the presence of the TNM stage (Table 8). It is probable that the TNM stage can represent tumor burden in a better way. When the TNM stage was applied, the other tumor burden indicators were not important any more. In contrast to the Korean study [6], age was a predictor of survival in both univariate and multivariate analyses. The independent predictive value of age may be attributed to its independence of tumor burden. These results further supported the rationality of the TNM staging system.

The clinical significance of tumor staging is not only for prognostication. More importantly, it is for treatment decision making. In the TNM staging, given the excellent prognosis of stage I disease, the tumor confined to T1/T2 probably cannot benefit from chemotherapy beyond radiation. The prognosis of T2N0M0 disease was slightly poorer than that of T1N0M0 disease. It is unknown whether there is any room for improvement in the management of T2 disease, so we retained the stages IA and IB. The role of chemotherapy in the management of stage II disease needs to be evaluated. A combined chemo-radiotherapy may be preferable for stage III disease. Neck radiation may be appropriate for N1/N2 disease. High dose chemotherapy and HSCT should be considered for stage IV N-NKTCL where radiation is inapplicable. Of course, these recommendations need to be validated in further investigations.

The TNM staging system was designed for N-NKTCL only. An independent staging method is necessary for E-NKTCL, as E-NKTCL is different from N-NKTCL in the clinical features, treatment approaches, prognostic factors, and clinical outcomes [1, 7]. In the current study, only 42 (10.4%) out of 405 cases were E-NKTCL, making it difficult to establish a staging system. In addition, modern imaging technology showed that most if not all E-NKTCLs are associated with occult nasal primaries [34], indicating that these cases are actually advanced stage N-NKTCL. If the occult nasal primaries are confirmed by careful examination, the application of TNM staging system is reasonable.

Limitations are inevitable due to the retrospective nature of the current study. To reduce confounders, we employed stringent exclusion criteria before the analysis. As a result, up to 134 patients were excluded. Even so, heterogeneity was present. Various chemotherapy regimens were used, the short-term efficacy of which in deed had some difference. The ASP-containing regimens generally showed better short-term efficacy than non-ASP-containing regimens. However, there is no evidence that one regimen is superior to another in improving OS. Besides, chemotherapy is not a curative treatment for this disease. Therefore, we think that the influence of the treatment heterogeneity was not negligible, but was insignificant. The proposed T classifications for tumor originating from the oral cavity, the oropharynx, and the hypopharynx are somewhat arbitrary due to the extremely low incidence and require optimization in the future when more data are available. Given the rarity of this disease, a multicenter prospective validation of the new staging system is warranted.

In the present study, we established a TNM staging system for N-NKTCL. The local/local-regional N-NKTCLs were classified into stages I to III according to the extent of primary tumor and RLNM, while tumor with any lesion outside the primary site and regional lymph node was classified into stage IV. Survival analysis demonstrated the excellent performance of this staging system in prognosticating OS and PFS. Meanwhile, the TNM stage may supersede several currently used predictive factors, thereby simplifying the risk stratification for N-NKTCL. More importantly, the TNM staging system may have significant implications in treatment decision for this disease.

## Supporting Information

S1 File Patient characteristics. (DOCX)

S2 File The comparison of asparaginase- and non-asparaginase-containing chemotherapies in patients with NK/T-cell lymphoma. (DOCX)
