# Supplementary material for: A TNM Staging System for Nasal NK/T-Cell Lymphoma
Source: PLoS One. 2015 Jun 22;10(6):e0130984. doi: 10.1371/journal.pone.0130984 (PMC4476596; doi:10.1371/journal.pone.0130984)
Supplement: S1 File — (DOCX) [file pone.0130984.s001.docx]

**Table S1. Patient characteristics**

| **Characteristic** | **No. (% or range)** |
| --- | --- |
| **Age (year)**  ≤ 60  > 60 | 41 (9 – 80)  241 (88.9)  30 (11.1) |
| **Gender** |  |
| Male | 173 (63.8) |
| Female | 98 (36.2) |
| **B symptoms**  No  Yes | 138 (50.9)  133 (49.1) |
| **LDH level**  Normal  Higher than normal | 198 (73.1)  73 (26.9) |
| **ECOG PS**  0-1  2-4 | 247 (91.1)  24 (8.9) |
| **Local invasiveness**  No  Yes | 107 (39.5)  164 (60.5) |
| **Tumor extent** |  |
| Confined to the head and neck | 230 (84.9) |
| Distal metastasis | 41 (15.1) |
| **Regional lymph node involvement** |  |
| No | 215 (79.3) |
| One side | 31 (11.4) |
| Both sides | 25 (9.2) |
| **Splenomegaly**^*^  No  Yes | 172 (63.5)  99 (36.5) |
| **Pretreatment plasma EBV-DNA (copy/mL)**^†^  ≤ 500  > 500 | 48 (37.5)  80 (62.5) |
| **AA stage** |  |
| I | 190 (70.1) |
| II | 46 (17.0) |
| III | 9 (3.3) |
| IV | 26 (9.6) |

^*^Splenomegaly refers to splenic enlargement without abnormal density lesions and is not considered as tumor infiltration. ^†^Data were available in 128 patients. Pretreatment plasma EBV-DNA level of > 500 copies/mL was identified as an adverse prognostic factor in a previous study[[1](#_ENREF_1)].

Abbreviations: LDH, lactate dehydrogenase; ECOG PS, Eastern Cooperative Oncology Group performance status ; AA, Ann Arbor.

1. Wang ZY, Liu QF, Wang H, Jin J, Wang WH, et al. (2012) Clinical implications of plasma Epstein-Barr virus DNA in early-stage extranodal nasal-type NK/T-cell lymphoma patients receiving primary radiotherapy. Blood 120: 2003-2010.
