# Supplementary material for: A TNM Staging System for Nasal NK/T-Cell Lymphoma
Source: PLoS One. 2015 Jun 22;10(6):e0130984. doi: 10.1371/journal.pone.0130984 (PMC4476596; doi:10.1371/journal.pone.0130984)
Supplement: S2 File — (DOCX) [file pone.0130984.s002.docx]

## The contemporary comparison of asparaginase- and non-asparaginase-containing chemotherapies in patients with NK/T-cell lymphoma

The asparaginase (ASP)-containing chemotherapies have been widely used in Sun Yat-sen University Cancer Center since June 2008, while non-ASP-containing chemotherapies were mainly used before this time. To avoid the time bias, the data of patients treated between June 2008 and November 2013 were analyzed separately. The complete remission (CR) rate, overall response rate (ORR), overall survival (OS), and progression-free survival (PFS) between the ASP- and non-ASP-containing groups were compared.

In this period, 78 and 79 patients were treated with front-line ASP- and non-ASP-containing chemotherapies, respectively. After the front-line chemotherapy, there were 46 CRs (59.0%) and 24 PRs (30.8%) in the ASP-containing group, and 29 CRs (36.7%) and 24 PRs (30.4%) in the non-ASP-containing group. Both CR rate (*P* < 0.001) and ORR (*P* = 0.001) were significant higher in the ASP-containing group. The OS was marginally superior (*P* = 0.057) (Fig. 1A), while the PFS was significantly better in the ASP-containing group (*P* = 0.003) (Fig. 1B).





Figure 1. The OS curves (A) and PFS curves (B) of patients in the ASP- and non-ASP-containing groups.
